# Acute and 28-Day Repeated-Dose Oral Toxicity Assessment of *Abelmoschus moschatus* Medik. in Healthy Wistar Rats

**DOI:** 10.1155/2020/1359050

**Published:** 2020-06-19

**Authors:** Sachinthi S. Amarasiri, Anoja P. Attanayake, Liyanagae D. A. M. Arawwawala, Kamani A. P. W. Jayatilaka, Lakmini K. B. Mudduwa

**Affiliations:** ^1^Department of Medical Laboratory Science, Faculty of Allied Health Sciences, University of Ruhuna, Galle, Sri Lanka; ^2^Department of Biochemistry, Faculty of Medicine, University of Ruhuna, Galle, Sri Lanka; ^3^Industrial Technology Institute, Colombo, Sri Lanka; ^4^Department of Pathology, Faculty of Medicine, University of Ruhuna, Galle, Sri Lanka

## Abstract

*Abelmoschus moschatus* Medik. (family: Malvaceae) has a long history of being used as a folk medicine in Sri Lanka. Despite the therapeutic use of this plant in traditional medicine, leaves of *A. moschatus* have not been subjected to scientific evaluation of toxicity/adverse effects *in vivo*. Thus, the present study was aimed to assess the acute and 28-day repeated-dose oral toxic effects of hexane (55 mg/kg), ethyl acetate (75 mg/kg), butanol (60 mg/kg), and aqueous (140 mg/kg) leaf extracts of *A. moschatus* in Wistar rats. Furthermore, identification of phytochemical constituents and determination of *in vitro* total antioxidant activity of the selected leaf extracts of *A. moschatus* were carried out. Repeated-dose oral administration of hexane and aqueous plant extracts produced no significant changes in the hematological profile and in selected biochemical parameters compared to the untreated healthy rats (*p* > 0.05). The administration of ethyl acetate and butanol extracts resulted in significant changes in some of the hematological parameters (*p* < 0.05), whereas biochemical parameters were not changed (*p* > 0.05). No significant changes in the relative organ weight of treated rats were observed (*p* > 0.05) except in the kidneys of Wistar rats treated with the ethyl acetate extract of *A. moschatus* (*p* < 0.05). Normal morphology with no signs of hemorrhages, necrosis, or inflammatory cell infiltrations was observed in the vital organs selected during the assessment of histopathology on H and E-stained tissue sections upon the treatment of selected extracts. Alkaloids were absent in the selected leaf extracts excluding the health risk for harmful alkaloids. The highest total antioxidant activity was reported in the butanol extract. In conclusion, the hexane and aqueous extracts of *A. moschatus* were completely nontoxic, whereas butanol and ethyl acetate extracts showed statistically significant changes in some hematological parameters and in relative organ weight of kidneys in healthy Wistar rats.

## 1. Introduction

A revival of interest in the use of phytomedicines has emerged worldwide for the management of a variety of diseases [1, 2]. Easy availability, low cost, efficacy, and fewer side effects account for this gained popularity towards ethnomedicines [3–6]. Despite the growing demand, the safety of herbal medicines is still a matter of concern. Sparse evidence is available on their bioactivities and toxic phytoconstituents for the majority of the herbal medicines in use. Plants may produce toxic secondary metabolites as natural defenses against adverse conditions [1]. These specialized secondary metabolites serve as signaling hormones, providing resistance against pests and diseases, attracting pollinators, and defending against herbivorous insects and pathogenic microorganisms [1, 7–9]. The secondary metabolites in plants have documented for their potency to modulate and modify biological activities in humans [10]. The functions of signaling molecules, neuropeptides, hormones, and neurotransmitters in the human body could be antagonized by the phytochemicals like alkaloids, flavonoids, terpenoids, and saponins present in medicinal plants [1, 11, 12]. Several alkaloids have shown agonistic activity on neurotransmitter systems whereas some terpenoids have shown inhibitory properties against mammalian cholinesterase. Similarly, aristolochic acid present in Aristolochia species has identified as a phytochemical toxicant involved in the development of nephropathies and carcinogenesis [1]. Pyrrolizidine alkaloids are another category found in more than 12 higher plant families which include toxic, carcinogenic, and teratogenic phytochemicals [13]. However, the potential toxicity of phytomedicines has become a serious health issue in the use of traditional medicine in many cultures across the world [10]. Hence, systematic scientific evaluation of toxicity in traditional herbal medicines is crucial, prior to the development of commercially viable drugs/dietary supplements and to incorporate in long-term treatments. Moreover, identification of toxic plant extracts or toxic compounds in medicinal plants would be extremely important since it would facilitate modification or rejection of possible toxicants at the early stages in the process of drug development from medicinal plants [1].

*Abelmoschus moschatus* Medik., commonly known as Kapukinissa (English name: Musk mallow, family: Malvaceae), is a medicinal plant with particular interest in the treatment of variety of diseases in traditional Ayurvedic practice in Sri Lanka [14]. Almost each part of the plant is used in traditional remedies in the forms of tinctures, decoctions, or pastes. Seeds are used in the treatment of fevers, intestinal disorders, urinary discharge, nervous debility, hysteria, skin diseases, and gonorrhea, etc. [14, 15]. Roots and leaves are used in the treatment of gonorrhea whereas flower infusion is used as a contraceptive [15]. The hydroalcoholic extract of the whole plant has demonstrated potent antilithiatic activity *in vivo* [16]. Seeds and leaves of the plant have been reported for diuretic, antioxidant, antiproliferative, and antimicrobial activities whereas different solvent extracts of the seeds have shown hepatoprotective, memory strengthening, and antiageing properties [16]. Several studies have revealed that different seed extracts as well as the seed oil of *A. moschatus* were free of toxic effects and safe to be used for edible purposes [16, 17]. Despite the use in traditional medicine applications, scientific evaluation of toxic effects of the leaves of *A. moschatus* has not been reported to date. The outcome of the study may fill the gap of the aforementioned bioactivities and incorporation of this plant in developing novel therapeutic agents for clinical use while providing some additional information on phytoconstituents. Herein, we assessed the acute and 28-day repeated-dose oral toxic effects of selected leaf extracts of *A. moschatus* in healthy Wistar rats. Further, qualitative analysis of phytoconstituents and determination of total antioxidant activity in the extracts of *A. moschatus* leaves were also carried out.

## 2. Materials and Methods

### 2.1. Chemicals, Reagents, and Instruments

Gallic acid, quercetin, 2,2-diphenyl-2-picrylhydrazyl, dichloromethane, and polyvinylpyrrolidone (PVP) were purchased from Sigma Chemical Co, USA, and were used in *in vitro* and *in vivo* experiments. All other chemicals and solvents were of analytical grade and were used without any purification.

A rotatory evaporator (Buchi, B-480, UK) was used for the efficient removal of solvents from plant extracts. A microplate reader (SpectraMax Plus, USA) was used in the determination of the total antioxidant activity of the selected medicinal plant extracts. A UV-visible, double-beam spectrophotometer (UV-1800 Shimadzu, USA) was used in the spectrophotometric bioassays. Hematological parameters were analyzed using an automated hematology analyzer (Mindray BC 5150, China). Tissue processor (Shandon, UK), microtome (Thermo Fisher, Germany), and microscope (Olympus CX 21, Japan) were used in the preparation and evaluation of hematoxylin and eosin- (H and E-) stained tissue sections.

### 2.2. Collection and Authentication of Plant Material

Leaves of *A. moschatus* were collected in September 2018, from the natural habitats in the Western province (6° 55′ 54.98″ N *× *79° 50′ 52.01″ E), Sri Lanka. The botanical identity of the plant was confirmed by comparing with the authentic samples at the National Herbarium, Royal Botanical Gardens, Peradeniya, Sri Lanka. A voucher specimen of the plant was deposited at the Department of Biochemistry, Faculty of Medicine, University of Ruhuna (voucher specimen number: PG/2016/55/01).

### 2.3. Preparation of Plant Extracts

Leaves of *A. moschatus* were dried at 40°C to a constant weight and coarsely ground. Powdered plant material was sequentially extracted with solvents in the order of increasing polarity, i.e., hexane, ethyl acetate, butanol, and water, by the Soxhlet extraction method. Solvents in hexane, ethyl acetate, and butanol extracts were removed from the extracts by evaporation under reduced pressure, in a rotary evaporator until two-thirds of the initial volume was removed. The resulting semisolid masses were vacuum-dried using a vacuum oven. The concentrated aqueous extract was finally freeze-dried at −40°C to obtain the lyophilized powder of the selected extract. The percentage yields of the hexane, ethyl acetate, butanol, and aqueous extracts were 4.29%, 5.76%, 4.53%, and 11.40%, respectively.

### 2.4. Assessment of Toxic Effects of the Selected Extracts of *Abelmoschus moschatus*

#### 2.4.1. Selection of Doses of Plant Extracts

The extracted plant materials were dissolved in a relevant vehicle for the preparation of the human equivalent therapeutic dose in rats. The hexane and ethyl acetate extracts were dissolved in corn oil (vehicle 1), and the butanol extract was dissolved in 3% polyvinylpyrrolidone (vehicle 2). Accordingly, the equivalent human therapeutic doses of the selected extracts of *A. moschatus* were as follows: hexane (55 mg/kg), ethyl acetate (75 mg/kg), butanol (60 mg/kg), and aqueous (140 mg/kg).

#### 2.4.2. Experimental Animals

Healthy male and female rats of Wistar strain (150 ± 25 g, 10–12 weeks of age), purchased from the Medical Research Institute, Colombo, Sri Lanka, were used in the experiments. The animals were housed in standard environmental conditions at the animal house of the Faculty of Medicine, University of Ruhuna, Sri Lanka. They were maintained on a standard laboratory diet of pellets and water *ad libitum*. The rats were allowed to acclimatize for a period of seven days under standard environmental conditions before the commencement of experiments. Ethical clearance was obtained from the Ethical Review Committee, Faculty of Medicine, University of Ruhuna, Sri Lanka (14.12.2015:3.1).

#### 2.4.3. Assessment of Acute Toxicity

Acute toxicity testing was performed for the selected plant extracts following the Organization for Economic Cooperation and Development (OECD) guideline 420, fixed-dose procedure [18].

Healthy Wistar rats were divided into four main groups, considering the average weight of animals. Each group consisted of ten animals including five male rats and five female rats. Group 1 rats, administered with equivalent volumes of distilled water once orally, served as the normal control. Group 2 and 3 rats were administered with the equivalent volumes of vehicle 1 (corn oil) and vehicle 2 (PVP), respectively. Animals of Group 4 were further divided into four subgroups (a–d; *n* = 5/sex/subgroup), and the hexane, ethyl acetate, butanol, and aqueous extracts of *A. moschatus* were orally administered. The animals were observed for three hours and then daily for 14 days for signs of toxicity such as changes in skin, fur, eyes, and mucous membranes, changes in respiration, occurrence of tremors, convulsions, salivation, diarrhea, lethargy, sleep, and coma.

#### 2.4.4. Assessment of 28-Day Repeated-Dose Oral Toxicity

Healthy Wistar rats were allotted to four groups, considering the average weight of animals. Each group consisted of ten animals including five male rats and five female rats. Group 1 rats, served as the untreated healthy control group, received distilled water daily. Group 2 and 3 were administered with the equivalent volumes of vehicle 1 (corn oil) and vehicle 2 (PVP), respectively. Group 4 consisted of four subgroups (a–d; *n* = 5/sex/subgroup), and each subgroup received the selected plant extracts (hexane, ethyl acetate, butanol, and aqueous extracts) of *A. moschatus* at the optimum effective dose (hexane: 55 mg/kg, ethyl acetate: 75 mg/kg, butanol: 60 mg/kg, and aqueous: 140 mg/kg; human equivalent therapeutic dose in rats, respectively) daily for 28 consecutive days. All animals were observed daily for clinical signs and mortality once before treatment, during treatment, and up to 4 h after treatment throughout the study period.

The body weight, consumption of food, and intake of water of each rat were assessed before the commencement of dosing, throughout the experimental period (28 days), and recorded at weekly intervals. The consumption of food and intake of water were calculated daily from the quantity of food, water supplied, and the amount remaining after 24 h.

The animals were sacrificed at the end of the study period of 28 days. Blood samples with a volume of 3.0 mL were collected by cardiac puncture for biochemical and hematological assessments. The heart, lung, small intestine, liver, spleen, and kidneys were excised for the assessment of the relative weight of organs and histopathological changes on H and E-stained sections.

#### 2.4.5. Assessment of Biochemical Parameters

Biochemical parameters including serum concentration of glucose [19], total cholesterol [20], and triacylglycerol [21] were estimated using spectrophotometric enzyme assay kits. Serum activity of alkaline phosphatase (ALP; EC 3.1.3.1) [22], alanine aminotransferase (ALT; EC 2.6.1.2) [23], aspartate aminotransferase (AST; EC 2.6.1.1) [24], and gamma-glutamyl transferase (*γ*-GT; EC 2.3.2.2) [25] were estimated. Serum concentrations of creatinine [26], blood urea nitrogen (BUN) [27], and total protein [28] were estimated using spectrophotometric assay kits.

#### 2.4.6. Assessment of Hematological Parameters

Hematological analysis was performed using an automated hematology analyzer. Hemoglobin concentration, total red blood cell count, platelet count, red cell indices including packed cell volume, mean corpuscular volume, mean corpuscular hemoglobin, and mean corpuscular hemoglobin concentration, total white blood cell count, and white blood cell differential counts of the blood samples were measured.

#### 2.4.7. Relative Weight of Organs

The relative organ weight of heart, lung, small intestine, liver, spleen, and kidneys of each animal was calculated by dividing the weight of the organ by the body weight of the animal as follows:

(1) $$\begin{array}{c} \text{relative organ weight }=\frac{\text{organ weight }\times 100\text{\% }}{\text{body weight on the day the animal was sacrificed}}. \end{array}$$

#### 2.4.8. Histology of Body Tissues

The heart, small intestine, liver, spleen, and kidney tissues were fixed in 10% buffered formalin in labeled bottles. H and E-stained tissue sections of the experimental animals were prepared for the assessment of histopathology. The tissue sections were examined under a light microscope to detect pathological alterations in the vital organs upon the treatment with plant extracts. Features of cell injury, cellular vacuolization, pyknosis, hemorrhage, and the presence of inflammatory cell infiltrations were searched on H and E-stained sections. The observations were confirmed by a consultant histopathologist.

### 2.5. Preliminary Phytochemical Screening

The presence of bioactive phytoconstituents including phenolic compounds, tannins, flavonoids, steroid glycosides, alkaloids, terpenoids, coumarins, and saponins was tested in the selected extracts of *A. moschatus* according to the standard protocol [29].

### 2.6. Development of Thin-Layer Chromatography Fingerprints

Thin-layer chromatography (TLC) profiles were developed for the selected extracts considering the polarity of solvents. The solvent systems which showed fine separations with a maximum number of components were selected for the development of TLC fingerprint.

### 2.7. Determination of Total Polyphenol Content

Total polyphenol content in the selected plant extracts was estimated using the Folin–Ciocalteu method with some modifications [30]. The plant extract (2 mg) in the form of a semisolid mass (hexane, ethyl acetate, and butanol extracts) or the lyophilized powder (aqueous extract) was dissolved in dimethyl sulfoxide (40–60 *μ*L) followed by 70% methanol to prepare 2 mg/mL solution. The solution was then diluted to prepare a dilution series (x2) in 70% methanol (1.0, 0.5, 0.25, and 0.125 mg/mL). Freshly prepared 10% Folin–Ciocalteu reagent (110 *μ*L) was added to the microplate and preplate reading was taken at 765 nm wavelength in the microplate reader. A Na_2_CO_3_ solution (1%, 70 *μ*L) was added to the mixture, and the plate was incubated at 25°C for 30 minutes. The absorbance of the resultant solution was measured at 765 nm against a distilled water blank in the microplate reader.

Quantification was done with respect to the standard curve of gallic acid in a range of 0–1 mg/mL (*y* = 3.2256*x* + 0.0121). The results are expressed in gallic acid equivalents of the dry weight (mg GAE/g).

### 2.8. Determination of Total Flavonoid Content

The total flavonoid content of plant extracts was determined by the method of Siddhuraju and Becker [31]. The plant extract (2 mg) in the form of semisolid mass (hexane, ethyl acetate, and butanol extracts) or lyophilized powder (aqueous extract) was initially dissolved in dimethyl sulfoxide (40–60 *μ*L) and then diluted in 70% methanol to prepare 2 mg/mL solution. The solution was then diluted to prepare a dilution series (x2) in 70% methanol (1.0, 0.5, 0.25, and 0.125 mg/mL). Similarly, a dilution series of the reference compound quercetin was prepared in methanol (1 mg/mL in methanol). The sample/reference compound (100 *μ*L) was added to the microplate and preplate reading was taken at 415 nm wavelength in the microplate reader. AlCl_3_ solution (2%, 100 *μ*L) was then added and the plate was incubated at 25°C for 10 minutes. The absorbance of the samples/reference compound was measured at 415 nm at the end of the incubation period. Methanol was used as the blank.

The flavonoid content was calculated using a standard of quercetin solution in the range of 0–125 *μ*g/mL. The results are expressed as mg quercetin equivalent/g of extract.

### 2.9. Determination of DPPH Scavenging Activity

The assay was performed according to the method of Blois [32]. The semisolid mass (hexane, ethyl acetate, and butanol extracts) or the lyophilized powder (aqueous extract) of the plant extract (2 mg) was initially dissolved in dimethyl sulfoxide (40–60 *μ*L) and then diluted in methanol to prepare 2 mg/mL solution. The solution was then diluted to prepare a dilution series (x2) in methanol (1.0, 0.5, 0.25, 0.125, and 0.0625 mg/mL). 6-Hydroxy-2,5,7,8-tetramethylchouroman-2-carboxylic acid (Trolox) (1 mg/mL in PBS) was used as the reference compound and it was diluted to prepare a dilution series (x2) in methanol (0.5, 0.25, 0.125, and 0.0625 mg/mL). Methanol (90 *μ*L) and sample/reference compound/methanol as the control (50 *μ*L) were added to the microplate and preplate reading was taken at 517 nm against the methanol blank. DPPH (20 mg in 100 mL of methanol) (60 *μ*L) was then added to the plate and incubated in dark at 25°C for 10 minutes. The absorbance of the sample/reference compound/control was measured at 517 nm at the end of the incubation period against the methanol blank. The antioxidant activity is expressed in terms of IC_50_ (concentration of the sample/reference compound at 50% inhibition).

Percentage DPPH radical scavenging activity was calculated as follows:

(2) $$\begin{array}{c} {\text{A}}_{\text{control}}\;=\text{ absorbance of the control,}\\ {\text{A}}_{\text{sample}}\;=\;\frac{\text{absorbance of the plant extract}}{\text{reference compound}},\\ \text{percentage of DPPH radical scavenging activity }=\frac{\;\left({\text{A}}_{\text{control}}{\;-\text{ A}}_{\text{sample}}\right)\times 100\text{\%}}{{\text{A}}_{\text{control}}}. \end{array}$$

### 2.10. Statistical Analysis

Data were statistically analyzed using SPSS software 22.0. Quantitative data were expressed as mean ± SEM. One-way ANOVA followed by Dunnet's post hoc test was used for multiple comparisons, and the values of *p* < 0.05 were considered statistically significant.

## 3. Results and Discussion

The potential toxic effects of several Sri Lankan medicinal plants detailed in traditional pharmacopoeias have been reported using *in vitro* and *in vivo* protocols. The seeds of *Holarrhena antidysenterica* (L.) Br. (family: Apocynaceae, common name: Kelinda), *Crotalaria juncea L*. (family: Leguminosae, common name: Hana), *Crotalaria verrucosa L*. (family Leguminosae, common name: Nil-andanahiriya) and bark, flowers, and seeds of *Cassia auriculata L*. (family: Leguminosae, common name: Ranawara) are few such examples that showed toxic effects due to the presence of pyrrolizidine alkaloids. Assessment of toxicity on animal models further substantiated possible liver, pulmonary, and renal toxicity of these medicinal plants in experimental rats producing histopathological changes [33, 34]. However, the potential toxicity of *C. auriculata* is critical since infusions of the flowers are a popular diuretic as well as a beverage (Ranawara tea) used by Sri Lankans [33]. In addition, Arseculeratne et al. [34] have identified five more plant species commonly used in Sri Lankan traditional medicine, namely, *Hemidesmus indicus* (stem), *Withania somnifera* (whole plant), *Madhuca longifolia* (seed), *Terminalia chebula* (fruit), *Aegle marmelos* (fruit) as negative for pyrrolizidine alkaloids, however, causing varying degrees of damage to hepatic, pulmonary, and renal tissues. These findings suggest the use of a wide range of medicinal plants with certain toxic substances in traditional remedies in Sri Lanka. Hence, there is an urgent necessity for conducting toxicity studies for medicinal plants that are widely used in traditional medicine in a suitable animal model.

### 3.1. Assessment of Toxic Effects of the Selected Extracts of *Abelmoschus moschatus*

Laboratory animals are widely used for the scientific evaluation of the safety of novel therapeutics prior to clinical trials [35]. The whole animal toxicity presumed to be closely related to human toxicity since the systems incorporate the pharmacokinetic nature of the test substance when administered by a similar route to its intended use [1]. However, rodent models still remain the gold standard in toxicity assessments [36]. A better extrapolation has been reported between rats and humans than in mice and humans [37]. Hence, toxicological assessment of leaf extracts of *A. moschatus* was carried out in the Wistar rat model.

#### 3.1.1. Assessment of Acute Toxicity

The administration of a single oral dose of plant extracts at the therapeutic dose induced neither mortality nor treatment-related signs of toxicity in experimental animals throughout the study period of 14 days. Further, body weight, consumption of food, and intake of water were not affected by the administration of plant extracts. In addition, the selected extracts of *A. moschatus* produced no adverse effects on experimental rats, and therefore, the human equivalent therapeutic dose was used in the 28-day repeated-dose oral toxicity assessment in the same animal model.

However, a 14-day behavioral toxicity study is inadequate to confirm the complete toxicity profile of a therapeutic agent [38]. This could not sufficient to determine the toxicity changes associated with long-term administration of herbal extracts in folk medicine [39]. Thus, long-term studies are essential for safety assessment of natural extracts. Accordingly, 28-day repeated-dose oral toxicity study was carried out in the experimental animals following OECD guidelines [40].

#### 3.1.2. Assessment of 28-Day Repeated-Dose Oral Toxicity

**Figure 1:**
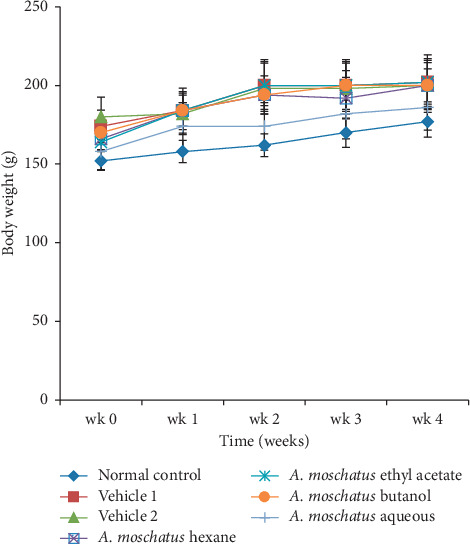
Change in body weight of Wistar rats administered with the hexane, ethyl acetate, butanol, and aqueous extracts of *Abelmoschus moschatus* during a 28-day repeated-dose oral toxicity study (*n* = 10/group). Each point represents mean ± SEM. No significant difference was observed in test group rats compared to the normal control (*p* > 0.05).

*(1) Body Weight, Consumption of Food, and Intake of Water*. The change in body weight of experimental rats treated with different extracts of *A. moschatus* during the intervention is shown in Figure 1. Change in the gain of body weight is considered as an early sign of toxicity upon an administration of a new drug. A loss or abnormal gain in body weight could be detected if rats are exposed to potential toxic substances [4, 41, 42]. Further, a significant loss of body weight is considered to be one of the most sensitive indicators of deteriorating health status [43]. However, daily administration of the hexane, ethyl acetate, butanol, and aqueous extracts of *A. moschatus* over a period of 28 days showed no significant changes in body weight gain in plant extract-treated rats compared to the normal control rats (*p* > 0.05). Furthermore, no significant changes were observed in the intake of water and consumption of food in the experimental rats treated with plant extracts compared to the untreated control group (*p* > 0.05). The findings suggested that the selected leaf extracts of *A. moschatus* had no adverse effects on the growth, metabolism, or health status of animals.

*(2) Biochemical Parameters*. When cells are exposed to toxic substances, a certain degree of damage to the cells may be caused by the toxic substances, resulting in cellular injury. This may lead to alterations in cellular permeability of the injured cells triggering the release of biochemical substances into the bloodstream [41].  Table 1 shows the results of the biochemical assessment in different groups of experimental animals treated with plant extracts.

The kidney and the liver are the vital organs to show toxic effects upon exposure to potential toxic substances as these organs are primarily involved in the detoxification process [41, 42]. The renal toxicity is indicated by an increased concentration of creatinine and BUN in the bloodstream due to the poor clearance of these waste products by the impaired kidneys [42, 44, 45]. Treatment with the extracts of *A. moschatus* did not change serum creatinine and BUN concentrations significantly compared to the normal control group, indicating no significant changes in the kidney function after the administration of plant extracts (*p* > 0.05). Therefore, the results ruled out the toxic effects of the selected plant extracts on kidney functions.

An increased serum concentration of liver enzymes such as ALT and AST is a reliable indicator of liver toxicity. These changes occur in the blood due to changes in hepatic cellular permeability or necrosis and cellular injury [13, 46, 47]. The 28-day repeated-dose oral administration of the selected extracts of *A. moschatus* caused a nonsignificant decrease in the concentration of ALT and AST compared to the untreated control group (*p* > 0.05). These results suggest that the treatment with the plant extracts did not cause any toxic effect on the liver. But the treatment with butanol extract of *A. moschatus* resulted in significant changes in the AST values (*p* < 0.05). However, the differences have no clinical significance since the values were found within the normal physiological range for the species [48]. Elevated levels of *ɣ*-GT and ALP are indicators of hepatobiliary obstruction and cholestatic induction [13, 42]. Serum total protein is considered as a nonenzymatic marker of liver toxicity [42, 49]. The results of *ɣ*-GT, ALP, and total protein in the plant extract-treated rats are compatible with the normal control rats and the findings exclude the hepatotoxic effects of the selected plant extracts.

The fasting blood glucose values and lipid profile parameters were estimated in experimental rats for the assessment of metabolic states in relation to carbohydrate and lipid metabolism, respectively. Repeated oral administration of the plant extracts resulted in a decrease in the level of glucose in plant-treated rats with a significant reduction in the ethyl acetate extract-treated group (*p* < 0.05). A mild reduction in the serum levels of triglycerides was noticed in plant extract-treated rats compared to the untreated control group. However, no significant differences were observed in the concentrations of total cholesterol and triglycerides in rats treated with plant extracts (*p* > 0.05).

*(3) Hematological Parameters*. Hematopoietic system in animals serves as an important indicator of physiological and pathological status for both animals and humans [41]. Hence, full blood count testing was performed and the results are shown in Table 2.

An elevation in total white blood cell counts was noticed in all groups of experimental rats treated with the plant extracts compared to the untreated control group, even though the changes were not significant (*p* > 0.05). However, the observed increase in total white blood cell counts directly indicates the strengthening of the defense systems in experimental rats. These findings further suggest that *A. moschatus* may contain biologically active compounds that have the ability to boost the immune system through increasing the population of defensive white blood cells. Further, an increase in red blood cell count, packed cell volume, and hemoglobin concentration was observed in experimental rats treated with plant extracts. However, significant elevations were observed only with ethyl acetate and butanol extracts of *A. moschatus* (*p* < 0.05). An increment in red blood cell parameters in the test groups compared to the untreated control group implying an increase in erythropoiesis due to the treatment with plant extracts [47]. The administration of plant extracts showed a reduction in the counts of eosinophil and monocyte compared to the control animals. A significant reduction was observed in the aqueous extract of *A. moschatus*; however, the results were within the physiological range described for the species [48]. A reduction in these white cell parameters could be attributed to the potential anti-inflammatory properties of *A. moschatus* as reported previously [16]. A significant variation in platelet counts was noticed with the treatment of hexane and ethyl acetate extracts of *A. moschatus* compared to the control group (*p* < 0.05). However, the results were within the physiological range [48].

*(4) Relative Weight of Organs*. Relative organ weight is an index often used in toxicological evaluations [2]. It is considered as a more specific parameter than the absolute weight in the evaluation of the toxicity in animal models [42]. Generally, a reduction in the internal weight of an organ is considered as an indication of toxicity following an exposure to toxic substances [45]. The heart, liver, spleen, kidney, and lungs are the primary organs affected by the metabolic reactions induced by toxicants [10]. In the excised vital organs, no significant changes (*p* > 0.05) in the relative organ weight were observed compared to the untreated control group in all organs following treatment for 28 days except in the group of rats treated with the ethyl acetate extract of *A. moschatus* (Table 3). A significant decrease was observed in the relative weight of kidneys following the treatment with the ethyl acetate extract of *A. moschatus* (*p* < 0.05), suggesting the possible toxic effects in Wistar rats.

*(5) Histology on H and E-Stained Sections*. The assessment of histology plays an important role in the evaluation of treatment-related organ damage. A biopsy may provide useful information for the elimination of competing causes of tissue damage by correlation with known patterns of drug-induced toxic changes. For example, inflammatory cell infiltrates are more often associated with drug-induced liver injury, whereas ductular reaction or hepatocellular accumulation of iron is less likely to be present. Further, assessment of histopathology may provide information on the severity of the injury, which may inform the prognosis. The degree of necrosis, fibrosis, and presence of inflammatory cell infiltrates are few findings which may be useful in determining the degree of tissue toxicity. The absence of significant injury may denote the safe use of the medication. Moreover, a biopsy may provide clues to the mechanism of tissue injury. For example, microvesicular steatosis in liver tissues suggests mitochondrial injury, while zonal necrosis in the absence of inflammation suggests an accumulation of a toxic metabolite [50]. Hence, the assessment of histological features of different organs has been carried out in a number of preclinical and clinical studies for the determination of drug-mediated toxic and adverse effects [51].

**Figure 2:**
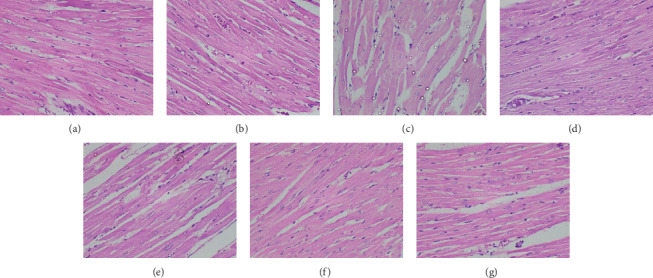
Photomicrographs of H and E-stained cardiac tissues of the different groups of experimental rats (×400). Healthy control group (a), vehicle 1: corn oil (b), vehicle 2: PVP (c), groups of animals treated with the hexane (d), ethyl acetate (e), butanol (f), and aqueous (g) extracts of *A. moschatus* at the equivalent therapeutic dose are shown. Repeated oral administration of the selected plant extracts for 28 consecutive days resulted in no significant adverse effects on the histomorphology of cardiac muscle and connective tissue. No signs of necrosis could be observed.

**Figure 3:**
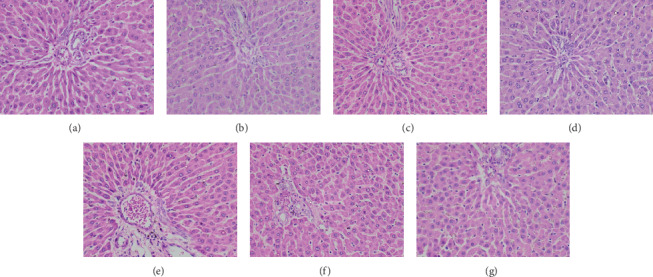
Photomicrographs of H and E-stained liver tissues of the different groups of experimental rats (×400). Healthy control group (a), vehicle 1: corn oil (b), vehicle 2: PVP (c), groups of animals treated with the hexane (d), ethyl acetate (e), butanol (f), and aqueous (g) extracts of *A. moschatus* at the equivalent therapeutic dose are shown. Repeated oral administration of the selected plant extracts for 28 consecutive days showed no significant adverse effects on the histomorphology of liver tissues. The cellular features of hepatocytes, sinusoids, and central vein in liver tissues of the experimental groups were similar to those in the untreated control group. The hepatocytes arranged in cords were normal. Early signs of cell injury, necrosis, congestion, or hemorrhage were not observed.

**Figure 4:**
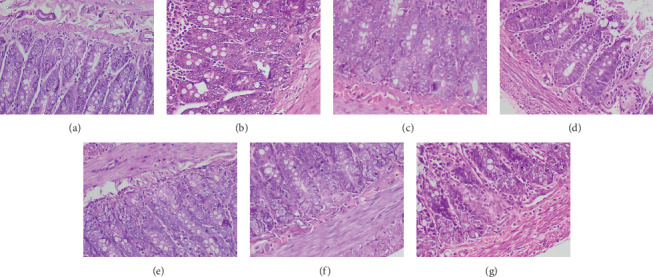
Photomicrographs of H and E-stained sections of the small intestine of the different groups of experimental rats (×400). Healthy control group (a), vehicle 1: corn oil (b), vehicle 2: PVP (c), groups of animals treated with the hexane (d), ethyl acetate (e), butanol (f), and aqueous (g) extracts of *A. moschatus* at the equivalent therapeutic dose are shown. Repeated oral administration of the selected plant extracts for 28 consecutive days showed no significant adverse effects on the histomorphology of tissues attributable to the treatment with plant extracts.

**Figure 5:**
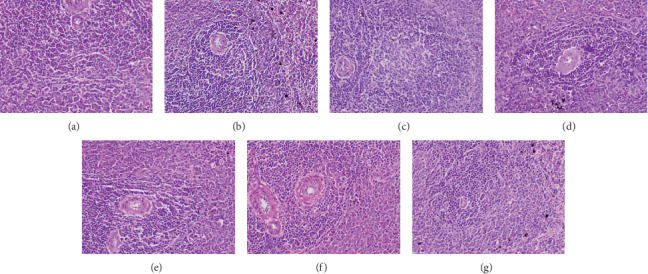
Photomicrographs of H and E-stained sections of spleen of the different groups of experimental rats (×400). Healthy control group (a), vehicle 1: corn oil (b), vehicle 2: PVP (c), groups of animals treated with the hexane (d), ethyl acetate (e), butanol (f), and aqueous (g) extracts of *A. moschatus* at the equivalent therapeutic dose are shown. Repeated oral administration of the selected plant extracts for 28 consecutive days showed no significant adverse effects on the histomorphology of spleen tissues attributable to the treatment with plant extracts.

**Figure 6:**
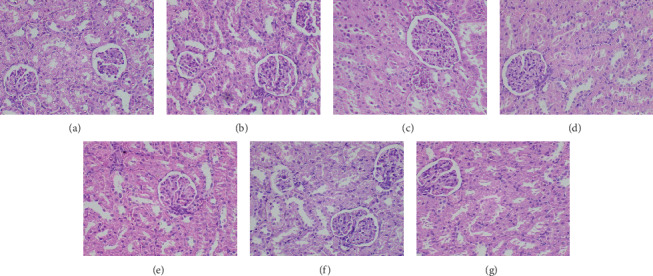
Photomicrographs of H and E-stained sections of kidney tissues of the different groups of experimental rats (×400). Healthy control group (a), vehicle 1: corn oil (b), vehicle 2: PVP (c), groups of animals treated with the hexane (d), ethyl acetate (e), butanol (f), and aqueous (g) extracts of *A. moschatus* at the equivalent therapeutic dose are shown. Repeated oral administration of the selected plant extracts for 28 consecutive days showed no significant adverse effects on the histomorphology of kidney tissues. Normal morphological architecture was observed with no signs of necrosis or inflammatory cell infiltration. The glomeruli, distal tubules, and proximal tubules appeared normal in all groups with no signs of glomerular congestion or tubular atrophy.

The histological findings of the present study corroborated the results of biochemical parameters. Compared to the untreated control group, light microscopic appearance of H and E-stained sections of the heart, liver, small intestine, spleen, and kidney of experimental rats showed normal structure following repeated administration of the plant extracts. Figures 2–6 shows the photomicrographs of the H and E-stained tissue sections of vital organs of the different groups of experimental animals. The cellular features of hepatocytes, sinusoids, and central vein in liver tissues of the experimental groups were similar to those in the untreated control group (Figure 3). The hepatocytes arranged in cords were normal. Early signs of cell injury, necrosis, congestion, or hemorrhage were not seen. The glomeruli, distal tubules, and proximal tubules in the kidney tissues appeared normal in all groups as shown in Figure 6. There was no glomerular congestion or tubular atrophy. Normal morphological architecture was observed with no signs of necrosis or inflammatory cell infiltrations in the kidney tissues. Even though mild hemorrhages were observed in kidney tissues of experimental animals treated with plant extracts, it was considered insignificant due to the presence of similar features in the untreated control group (Figure 6(a)). The cellular structures of cardiac muscle and connective tissues were normal (Figure 2). No signs of necrosis could be observed. Similarly, no abnormalities were observed in the spleen (Figure 5) and small intestine (Figure 4) of the rats following the administration of the plant extracts as compared with those of rats in the normal control group. These findings revealed that daily administration of the selected plant extracts for 28 days did not cause any detrimental changes or morphological disturbances to the rats or to their vital organs. Even though a significant reduction in the relative weight of kidneys was observed with the treatment of ethyl acetate extract of *A. moschatus*, no signs of toxicity were observed in the particular group in the assessment of histopathology.

### 3.2. Phytochemical Analysis

The findings of the preliminary phytochemical screening of the selected leaf extracts of *A. moschatus* are summarized in Table 4. The extracts of *A. moschatus* had varying levels of phenolic compounds in the descending order of aqueous > butanol > ethyl acetate > hexane; in contrast, coumarins and alkaloids were absent. The absence of alkaloids excludes the risk of harmful alkaloids such as pyrrolizidine alkaloids, and these findings are in agreement with Arseculeratne et al. [34]. Tannins, flavonoids, and terpenoids were present in both butanol and aqueous extracts whereas steroid glycosides and saponins were present only in the aqueous extract of *A. moschatus*.

### 3.3. Thin-Layer Chromatography Fingerprints

**Figure 7:**
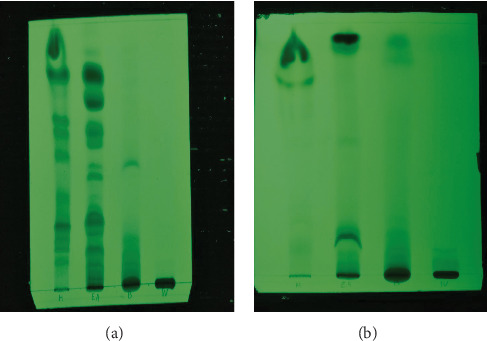
TLC profiles for hexane, ethyl acetate, butanol, and aqueous extracts of *Abelmoschus moschatus.* (a) TLC fingerprint under the solvent system: dichloromethane : cyclohexane : methanol (1 : 1  : 0.1) for improved separation of the nonpolar extracts (hexane and ethyl acetate). (b) TLC fingerprint under the solvent system: dichloromethane : cyclohexane : methanol : diethylamine (1 : 0.8 : 0.1 : 0.3) for improved separation of the polar extracts (butanol and water).

TLC profiles were developed for the selected extracts of *A. moschatus* in order to use as fingerprints of each respective extract. This was done using two solvent systems considering the polarity of extracts in order to improve the separation. Accordingly, the solvent systems made of dichloromethane: cyclohexane: methanol (1 : 1 : 0.1) and dichloromethane: cyclohexane: methanol: diethylamine (1 : 0.8 : 0.1 : 0.3) were developed for the proper separation of nonpolar and polar extracts, respectively.  Figure 7 shows the results of the analysis of TLC fingerprint profiles.

### 3.4. Total Polyphenol and Flavonoid Contents

Table 5 shows the results of total polyphenol content and total flavonoid content in the selected extracts of *A. moschatus*. The highest polyphenol content was found in the ethyl acetate extract whereas the lowest value was found in the hexane extract. The butanol extract reported the highest total flavonoid content while the aqueous extract reported the lowest values. However, the ethyl acetate extract showed relatively higher concentrations of both polyphenols and flavonoids than the other three extracts.

### 3.5. DPPH Scavenging Activity

Antioxidants in medicinal plants play a major role in the defense against oxidative damage caused by reactive oxygen species [7, 52]. Therefore, the total antioxidant activity of the four selected extracts of *A. moschatus* was assessed by DPPH radical scavenging assay. It is a simple, rapid, and relatively inexpensive assay, widely used in the evaluation of the antioxidant potential of aqueous or organic extracts with hydrophilic and lipophilic compounds [7, 53]. The total antioxidant activity is expressed in terms of IC_50_ (Table 5).

In DPPH radical scavenging assay, low IC_50_ values represent a high level of antioxidant activity and *vice versa* [54, 55]. The total antioxidant activity of the four selected extracts deviated in the descending order of butanol, ethyl acetate, aqueous, and hexane extracts of *A. moschatus*, respectively. These findings are corroborated with the total polyphenol content of the selected extracts showing similar variations in results with mild deviations. Further, these findings suggest that high polyphenol content in the ethyl acetate and butanol extracts might be attributed to the high total antioxidant activity *in vitro*.

Values are expressed as mean ± SD of three measurements in each group. Data are expressed as milligrams of gallic acid equivalents per gram of extract and milligrams of quercetin equivalents per gram of extract for total polyphenol content and total flavonoid contents, respectively. The total antioxidant capacity is expressed in terms of IC_50_ (*μ*g/mL); the concentration of the antioxidant required for a decrease in the percentage of inhibition is 50% of the initial DPPH concentration.

## 4. Conclusion

Findings of the present study revealed neither mortality nor behavioral changes in the acute and 28-day repeated dose oral toxicity assessment of hexane (55 mg/kg), ethyl acetate (75 mg/kg), butanol (60 mg/kg), and aqueous (140 mg/kg) plant extracts of *A. moschatus* in healthy Wistar rats. Based on the results of biochemical, hematological, and histopathological assessments, we conclude that 28-day repeated-dose oral administration of the hexane and aqueous extracts of *A. moschatus* at the equivalent human therapeutic doses to Wistar rats is completely safe. A significant increase in red blood cell count, hematocrit, and hemoglobin concentration was observed in experimental rats treated with ethyl acetate and butanol extracts of *A. moschatus*. Further, statistically significant changes in the concentration of fasting plasma glucose and AST activity were noted in the same groups; however, the values were found within the normal physiological range for the species. A significant reduction in the relative weight of kidneys was observed with the treatment of ethyl acetate extract of *A. moschatus*; however, no signs of toxicity were observed in the particular group of rats on H and E-stained sections of the kidney tissues. The absence of harmful alkaloids further substantiates the findings on 28-day repeated-dose toxicity study. DPPH assay revealed the free radical scavenging potential of the selected extracts which could be attributed to the presence of phytoconstituents such as polyphenols in the selected plant extracts. The butanol extract showed the highest antioxidant potential whereas the hexane extract showed the lowest.

## Figures and Tables

**Table 1:** Biochemical parameters in serum after treatment with selected plant extracts of *Abelmoschus moschatus* for 28 days.

	Normal control	Vehicle corn oil	Vehicle PVP	Hexane extract	Ethyl acetate extract	Butanol extract	Aqueous extract
BUN (mmol/L)	8.12 ± 0.50	6.89 ± 0.89	7.25 ± 0.49	7.84 ± 1.07	7.22 ± 0.49	8.34 ± 0.61	8.37 ± 0.27
Creatinine (*μ*mol/L)	66.39 ± 2.23	55.25 ± 3.51	56.75 ± 2.46	58.87 ± 1.71	54.10 ± 2.79	56.75 ± 2.43	55.47 ± 3.86
Fasting plasma glucose (mmol/L)	5.48 ± 0.14	4.50 ± 0.23	5.16 ± 0.44	4.85 ± 0.43	4.07 ± 0.22^a^	4.82 ± 0.41	4.89 ± 0.40
Total cholesterol (mmol/L)	1.73 ± 0.08	1.47 ± 0.16	1.56 ± 0.10	1.96 ± 0.08	1.51 ± 0.16	1.72 ± 0.10	1.92 ± 0.11
Triglycerides (mmol/L)	1.25 ± 0.09	0.86 ± 0.04^b^	0.88 ± 0.09a	1.00 ± 0.05	1.12 ± 0.11	0.95 ± 0.12	0.96 ± 0.08
Total protein (g/L)	76.11 ± 2.48	75.64 ± 3.61	80.00 ± 3.77	70.08 ± 2.22	70.56 ± 2.87	68.26 ± 1.24	68.65 ± 1.62
AST (U/L)	109.07 ± 12.02	76.7 ± 9.41	76.48 ± 7.11	101.28 ± 14.70	81.94 ± 3.93	66.00 ± 17.52a	85.56 ± 3.28
ALT (U/L)	50.17 ± 2.22	39.46 ± 2.77	38.99 ± 2.40	46.56 ± 6.11	41.61 ± 2.95	38.53 ± 6.66	43.18 ± 2.29
ALP (U/L)	187.74 ± 16.80	195.76 ± 16.10	183.40 ± 18.27	224.26 ± 17.20	189.68 ± 18.44	218.36 ± 17.44	214.12 ± 37.01
*ɣ*-GT (U/L)	6.76 ± 0.63	4.86 ± 1.25	4.75 ± 1.16	3.62 ± 0.43	5.21 ± 1.62	4.17 ± 0.56	4.75 ± 0.34

Values are expressed as mean ± SEM of ten animals in each group. Results are significant compared to the untreated control group at ^a^*p* < 0.05, ^b^*p* < 0.01, and ^c^*p* < 0.001. BUN: blood urea nitrogen; HDL-C: high-density cholesterol; LDL-C: low-density lipoprotein cholesterol; AST: aspartate aminotransferase; ALT: alanine aminotransferase; ALP: alkaline phosphatase; *γ*-GT: gamma-glutamyl transferase.

**Table 2:** Hematological parameters in whole blood after treatment with selected plant extracts of *Abelmoschus moschatus* for 28 days.

	Normal control	Vehicle corn oil	Vehicle PVP	Hexane extract	Ethyl acetate extract	Butanol extract	Aqueous extract
RBC (×106/*μ*L)	7.52 ± 0.14	7.97 ± 0.19	8.20 ± 0.25	8.26 ± 0.22	8.46 ± 0.11^a^	8.48 ± 0.17^a^	8.19 ± 0.18
Hb (g/dL)	13.81 ± 0.25	14.04 ± 0.10	13.86 ± 0.14	14.88 ± 0.47	15.06 ± 0.29^a^	14.38 ± 0.29	14.44 ± 0.23
PCV (%)	50.70 ± 1.03	53.74 ± 0.98	53.86 ± 1.56	53.10 ± 1.88	55.48 ± 0.84	56.16 ± 1.01^a^	53.84 ± 0.93
PLT (×103/*μ*L)	258.30 ± 17.83	240.00 ± 12.19	243.00 ± 18.23	337.00 ± 23.30^a^	188.40 ± 5.49^a^	275.20 ± 21.36	282.60 ± 12.57
WBC (per mm^3^)	2.40 ± 0.48	2.24 ± 0.48	2.72 ± 0.57	4.46 ± 0.70	4.00 ± 0.20	3.64 ± 0.38	4.58 ± 0.77
Neutrophils (%)	18.33 ± 1.32	18.20 ± 2.48	16.80 ± 2.44	18.60 ± 0.68	20.00 ± 3.78	21.00 ± 4.16	18.40 ± 2.42
Lymphocytes (%)	73.70 ± 2.34	79.00 ± 1.79	73.20 ± 2.22	73.80 ± 2.22	74.80 ± 3.17	71.00 ± 3.83	78.40 ± 2.50
Eosinophils (%)	1.80 ± 0.59	1.20 ± 0.20	1.20 ± 0.20	1.60 ± 0.68	0.80 ± 0.37	0.80 ± 0.20	1.20 ± 0.37
Basophils (%)	1.70 ± 0.26	3.00 ± 0.00	2.40 ± 0.40	1.80 ± 0.58	2.40 ± 0.75	2.20 ± 0.20	1.60 ± 0.51
Monocytes (%)	1.86 ± 0.34	1.20 ± 0.20	1.00 ± 0.00	0.60 ± 0.24	1.20 ± 0.37	1.40 ± 0.24	0.40 ± 0.40^a^
MCV (fL)	66.83 ± 0.27	66.88 ± 0.31	65.72 ± 0.17	65.76 ± 0.26	67.26 ± 0.26	66.60 ± 0.10	65.82 ± 0.41
MCH (pg)	18.13 ± 0.13	17.66 ± 0.22	17.56 ± 0.14	17.80 ± 0.10	18.22 ± 0.08	17.76 ± 0.08	17.82 ± 0.09
MCHC (g/dL)	27.23 ± 0.20	26.42 ± 0.23^a^	26.64 ± 0.12	27.04 ± 0.15	27.22 ± 0.11	26.78 ± 0.06	27.10 ± 0.14

Values are expressed as mean ± SEM of ten animals in each group. Results are significant compared to the untreated control group at ^a^*p* < 0.05, ^b^*p* < 0.01, and ^c^*p* < 0.001. RBC: total red blood cell count; Hb: hemoglobin concentration; PCV: packed cell volume; PLT: platelet count; WBC: total white blood cell count; MCV: mean corpuscular volume; MCH: mean corpuscular hemoglobin; MCHC: mean corpuscular hemoglobin concentration.

**Table 3:** Relative weight of organs (g/g BW) after treatment with selected plant extracts of *Abelmoschus moschatus* for 28 days.

	Normal control	Vehicle corn oil	Vehicle PVP	Hexane extract	Ethyl acetate extract	Butanol extract	Aqueous extract
Heart	0.33 ± 0.01	0.33 ± 0.01	0.35 ± 0.01	0.32 ± 0.01	0.32 ± 0.00	0.32 ± 0.01	0.34 ± 0.01
Liver	2.68 ± 0.03	2.60 ± 0.04	2.61 ± 0.04	2.62 ± 0.05	2.57 ± 0.05	2.74 ± 0.04	2.71 ± 0.07
Small intestine	1.78 ± 0.09	1.43 ± 0.12	1.73 ± 0.12	1.51 ± 0.07	1.52 ± 0.15	1.48 ± 0.09	1.48 ± 0.09
Lungs	0.54 ± 0.01	0.47 ± 0.02^a^	0.50 ± 0.02	0.51 ± 0.02	0.49 ± 0.00	0.49 ± 0.02	0.52 ± 0.01
Spleen	0.25 ± 0.00	0.24 ± 0.01	0.25 ± 0.01	0.24 ± 0.01	0.26 ± 0.01	0.24 ± 0.01	0.24 ± 0.01
Kidneys	0.59 ± 0.01	0.56 ± 0.01	0.55 ± 0.01	0.55 ± 0.01	0.52 ± 0.01^a^	0.54 ± 0.01	0.57 ± 0.01

Values are expressed as mean ± SEM of 10 animals in each group. Results are significant compared to the untreated control group at ^a^*p* < 0.05, ^b^*p* < 0.01, and ^c^*p* < 0.001.

**Table 4:** Phytoconstituents present in the selected extracts of *Abelmoschus moschatus*.

	Hexane extract	Ethyl acetate extract	Butanol extract	Aqueous extract
Phenolic compounds	+	++	+++	++++
Tannins	−	−	+	+
Flavonoids	−	−	++	+
Steroid glycosides	−	−	−	+
Coumarins	−	−	−	−
Terpenoids	−	−	+	+
Saponins	−	−	−	+
Alkaloids	−	−	−	−

The presence of phytoconstituents is expressed as + whereas the absence of phytoconstituents is expressed as −. The degree of presence is expressed as follows: +: present and in mild level; ++: present and in moderate level; +++: present and in higher level; ++++: present and in abundant level.

**Table 5:** Total polyphenol content, total flavonoid content, and total antioxidant potential of the leaf extracts of *Abelmoschus moschatus*.

Plant extract	Total polyphenol content (mg gallic acid/g of extract)	Total flavonoid content (mg quercetin/g of extract)	Total antioxidant capacity by DPPH assay; IC_50_ (*μ*g/mL)
Hexane	0.17 ± 0.07	56.82 ± 3.57	451.49 ± 17.95
Ethyl acetate	23.09 ± 0.09	54.81 ± 1.45	235.57 ± 4.71
Butanol	3.56 ± 0.19	61.52 ± 0.24	124.48 ± 1.15
Aqueous	0.89 ± 0.02	4.62 ± 0.02	267.70 ± 4.88

## Data Availability

The data used to support the findings of this study are included within the article.

## Abbreviations

ANOVA: Analysis of variance
ALP: Alkaline phosphatase
ALT: Alanine aminotransferase
AST: Aspartate aminotransferase
BUN: Blood urea nitrogen
DPPH: 2,2-Diphenyl-2-picrylhydrazyl
*γ*-GT: Gamma-glutamyl transferase
H and E: Hematoxylin and eosin
Hb: Hemoglobin concentration
HDL-C: High-density cholesterol
IC_50_: Concentration of the extract/reference compound at 50% inhibition
LDL-C: Low-density lipoprotein cholesterol
MCH: Mean corpuscular hemoglobin
MCHC: Mean corpuscular hemoglobin concentration
MCV: Mean corpuscular volume
OECD: Organization for Economic Cooperation and Development
PCV: Packed cell volume
PLT: Platelet count
PVP: Polyvinylpyrrolidone
RBC: Red blood cell count
SD: Standard deviation
SEM: Standard error of the mean
SPSS: Statistical Package for Social Sciences
TLC: Thin-layer chromatography
Trolox: 6-Hydroxy-2,5,7,8-tetramethylchroman-2-carboxylic acid
WBC: White blood cell count.
